# Gut Microbiota Dysbiosis Remodels the Lysine Acetylome of the Mouse Cecum in Early Life

**DOI:** 10.3390/biology14080917

**Published:** 2025-07-23

**Authors:** Yubing Zeng, Jinying Shen, Xuejia He, Fan Liu, Yi Wang, Yi Wang, Yanan Qiao, Pei Pei, Shan Wang

**Affiliations:** 1 Capital Institute of Pediatrics, Chinese Academy of Medical Sciences & Peking Union Medical College, Beijing 100020, China; awater007@sina.com (Y.Z.); wang_yii@163.com (Y.W.); 2 Beijing Municipal Key Laboratory of Child Development and Nutriomics, Capital Institute of Pediatrics, Beijing 100020, China; 13726256164@163.com (J.S.); 15628830436@163.com (X.H.); qiaoyanan5114@126.com (Y.Q.); 13661202592@163.com (P.P.); 3 Beijing Tongren Hospital, Capital Medical University, Beijing 100005, China; 4 Beijing Municipal Key Laboratory of Child Development and Nutriomics, Capital Institute of Pediatrics, Peking University Teaching Hospital, Beijing 100020, China; 5 Child Healthcare Center, Capital Center for Children’s Health, Capital Medical University, Beijing 100020, China; liufan5023@163.com; 6 Laboratory of Infection and Virology, Beijing Pediatric Research Institute, Beijing Children’s Hospital, Capital Medical University, National Center for Children’s Health, Beijing 100045, China; wangyiyvonne29@sina.com

**Keywords:** gut microbiota dysbiosis, mouse cecum, post-translational modification, acetylation, metabolism

## Abstract

The interaction between epigenetic mechanisms and the gut microbiome is potentially crucial for the development and maintenance of intestinal health. Lysine acetylation, an important post-translational modification, plays a complex and critical role in the epigenetic regulation of the host by the gut microbiota. However, there are currently no reports on how gut microbiota dysbiosis affects host physiology in early life through global lysine acetylation. In this study, we constructed a mouse model of gut microbiota dysbiosis using antibiotic cocktail therapy (ABX). Using liquid chromatography-tandem mass spectrometry (LC-MS/MS) in the cecum, we analyzed the cecal lysine acetylome and proteome. As a result, we profiled the lysine acetylation landscape of the cecum and identified a total of 16,579 acetylation sites from 5218 proteins. Differentially acetylated proteins (DAPs) are involved in various metabolic pathways, including the citrate cycle (TCA cycle), butanoate metabolism, pyruvate metabolism, glycolysis/gluconeogenesis, and fatty acid biosynthesis. Moreover, both glycolysis and gluconeogenesis are significantly enriched in acetylation and protein modifications. This study aimed to provide valuable insights into the epigenetic molecular mechanisms associated with host protein acetylation as influenced by early-life gut microbiota disturbances. It reveals potential therapeutic targets for metabolic disorders linked to gut microbiota dysbiosis, thereby establishing a theoretical foundation for the clinical prevention and treatment of diseases arising from such dysbiosis.

## Simple Summary

There is a close connection between gut microbiota and the host’s epigenetics. Lysine acetylation, as a common post-translational modification, plays a complex role in the epigenetic regulation of the host by gut microbiota. This study aimed to determine the impact of gut microbiota dysbiosis during early life on host epigenetic regulation. We used 3-week-old mice to simulate this critical window of gut microbiota development in early life. Our research reveals the lysine acetylation and proteomic characteristics of cecal tissue in a mouse model of gut microbiota dysbiosis during early life. Our findings indicate that dysbiosis of the early gut microbiota can disrupt host metabolism by regulating global protein acetylation.

## 1. Introduction

The gut microbiota and their hosts have coevolved in a symbiotic relationship, participating in essential physiological functions such as digestion, nutrient absorption, immune response, and maintenance of energy balance. These functions are crucial for safeguarding the host’s homeostatic environment [1,2]. The development of newborns and their associated microbiota are closely associated with breastfeeding [3]. Following the cessation of exclusive breastfeeding, infants begin to process external foods with their teeth. This introduction of solid foods alters the gut environment, promoting the emergence of bacterial communities that possess significant metabolic capabilities. The diversity of the gut microbiota continues to increase until at least three years of age [4]. At the microscopic level, the gut microbiota plays a pivotal role in regulating host physiological processes by producing various bioactive substances, including neurotransmitters, vitamins, and bile acids. These substances serve dual functions as metabolic signaling molecules and reactive substrates within the host system [5,6]. Microbial metabolites are absorbed by the intestinal lining and can interact with cells throughout the body via the bloodstream [2,7]. Collectively, these studies underscore the critical importance of the gut microbiota during early life stages for optimal host health.

In recent years, research on host epigenetic changes induced by the gut microbiota has revealed potential mechanisms underlying the interaction between epigenetics and gut microbes. It has been demonstrated that epigenetic regulation mediated by the gut microbiota constitutes a form of host-microbe interaction. Microbiome-induced epigenetic alterations include modifications to DNA and histones, chromatin accessibility, and the activity of noncoding RNA [2]. The interplay between epigenetic changes and the gut microbiota can be facilitated by metabolites derived from the microbiome, which act as substrates and cofactors for key epigenetic enzymes within the host. For example, folate is an essential nutrient synthesized by various symbiotic microorganisms, including beneficial bacteria such as Bifidobacteria and Lactobacilli. These microorganisms engage in one-carbon metabolism to produce S-adenosylmethionine (SAM), thereby influencing the methylation status of DNA or histones in the host [8]. Dysbiosis of the gut microbiota can modulate the levels of both the host transcriptome and the m6A epitranscriptome through bile acid metabolic pathways [9]. Typically, the gut microbiota generates short-chain fatty acids (SCFAs) via polysaccharide fermentation [10]. SCFAs provide nourishment to intestinal cells while maintaining an optimal pH within the gastrointestinal environment, thus preserving intestinal barrier integrity [11]. Furthermore, SCFAs inhibit the activity of host histone deacetylases (HDACs), leading to increased acetylation levels that reduce chromatin condensation and diminish transcriptional silencing [12]. Additionally, SCFAs have been shown to influence TET enzyme activity; when these enzymes target gene promoters, they can induce DNA demethylation and increase gene transcription [13]. Moreover, studies have revealed that the gut microbiota regulates butyrylation and propionylation processes in intestinal epithelial cells in mice; these two forms of epigenetic modification correlate with SCFA abundance [14].

Post-translational modifications (PTMs) of proteins are pivotal in regulating cellular functions. The dynamic alterations of these modifications play essential roles in disease development, developmental processes, and adaptive mechanisms. Numerous studies have highlighted that gut microbiota play a crucial role in regulating host epigenetics through the modulating of PTMs. For example, transcriptomic analyses and overall DNA methylation assessment of intestinal epithelial cells from GF mice demonstrate that microbial exposure leads to DNA hypomethylation and an upregulation of the antimicrobial and anti-inflammatory gene expression [15]. In the colons of mice treated with antibiotics, the levels of histone H3 and H4 crotonylation were significantly reduced, and the use of the HDAC inhibitor butyrate promoted histone crotonylation [16]. These studies indicate that the microbiota stimulate various types of histone modifications and modulate the activity of histone-modifying enzymes, thereby calibrate both local and extraintestinal chromatin. A well-established PTM process influenced by metabolism is histone acetylation [14]. Compared with other PTMs linked with metabolic activities, such as phosphorylation, acetylation is more prevalent in microorganisms [17]. In bacteria, both enzymatic and nonenzymatic acetylation mechanisms allow up to 40% of proteins to undergo acetylation [18,19]. In a mouse model with depleted gut microbiota, the levels of pan-Kac in cecal tissue were lower than those reported in specific pathogen-free (SPF) mice; a similar enrichment change was noted for H3K27ac [14]. Overall, the levels of histone H4 decreased relatively uniformly in epithelial cells isolated from the cecum and colon of germ-free female mice. Furthermore, experiments utilizing 13C-labeled fiber tracking revealed that the microbiota can convert dietary fiber into butyrate, which subsequently provides acetyl-CoA—a necessary cofactor for histone acetyltransferases (HATs) [20]. Additionally, studies have confirmed that acetylation is prevalent within key metabolic pathways associated with the human gut microbiota, particularly within glycolytic metabolism and SCFA production [21]. Consequently, investigating the relationship between lysine acetylation and the gut microbiome is vital for gaining deeper insights into the regulatory mechanisms governing the host–microbiome axis. However, there are currently no reports addressing how dysbiosis of the gut microbiota impacts host physiology during early life through global lysine acetylation.

In this study, we investigated the effects of antibiotic-induced gut microbiota dysbiosis on the lysine acetylome and proteomic profiles in mouse cecal tissues. Given the critical role of gut microbiota in host metabolic processes and epigenetic regulation, this study aimed to provide new insights into the molecular mechanisms linking microbial imbalances to host metabolic pathways, with a particular focus on the TCA cycle and butanoate metabolism.

## 2. Materials and Methods

### 2.1. Animals

This research involved male C57BL/6J mice, sourced from Jiangsu Jicui Yaokang Biotechnology Co., Ltd., located in Nanjing, Jiangsu, China. At the start of the experiment, the mice were 3 weeks old and had a weight ranging from 8 to 13 g. To prioritize their health, all mice included in the research were confirmed free from specific pathogens. During the experimental phase, the mice were kept in a regulated environment featuring a 12 h light/dark cycle. They had unrestricted access to food and water. Temperature and humidity levels were carefully monitored to ensure stability. Following a 3-day acclimatization period in their new environment, the mice were randomly divided into either the antibiotic (ABX) group or the control (CON) group (*n* = 8/group). The sample size was calculated considering a statistical power of 80% and α = 0.05. All experiments were performed in strict compliance with the applicable ethical guidelines. The research obtained endorsement from the Ethics Committee of the Capital Institute of Pediatrics on 10 November 2021 (DWLL2021016).

### 2.2. ABX Protocol

We followed the method for constructing antibiotic-induced gut microbiota in mice [22]. In the first week, antibiotics were administered via drinking water (ampicillin 1 g/L, vancomycin 0.5 g/L, neomycin 1 g/L, metronidazole 1 g/L), with the antibiotic solution being changed every two days. In the second week, antibiotics were administered orally on a daily basis (ampicillin 5 mg/mouse, vancomycin 5 mg/mouse, neomycin 5 mg/mouse, metronidazole 4 mg/mouse), diluted in saline, with metronidazole diluted using 0.1 M acetic acid. Finally, the intervention of the gut microbiota in the mice was determined through Qubit experiments. During the experiment, the body weight, water intake, and food consumption of the mice were measured every two days, and measurements were taken daily before and after gavage. The length of the mice from the tip of the nose to the genitalia and from the tip of the nose to the tip of the tail was measured once a week.

### 2.3. Hematoxylin–Eosin (HE) Staining

To assess the damage to cecal tissue induced by antibiotic-induced dysbiosis in three mice from each group, cecum tissues were harvested after euthanizing the mice. The fecal contents were thoroughly rinsed from the cecum while ensuring the preservation of the epithelial structure. The tissues were preserved in formaldehyde for a period of 24 h, subsequently dehydrated using alcohol, and then embedded in paraffin. Once the paraffin blocks were prepared, they were cut into slices of 4 µm thickness and stained with HE. Before washing with distilled water, the sections underwent deparaffinization and dehydration using xylene and ethanol. For the HE staining procedure, the sections received treatment with hematoxylin solution (Servicebio, Wuhan, China, G1003), followed by differentiation and bluing solutions, and were then fixed with ethanol and stained using eosin. Afterward, the sections were dehydrated again, immersed in xylene, and finally sealed with neutral gum.

### 2.4. Immunohistochemical (IHC) Staining

Antigens were repaired. The sections were baked, dewaxed, dehydrated, and blocked with 5% goat serum. They were then incubated with primary antibodies P300 (dilution 1:500, CST, Danvers, MA, USA), SIRT1 (dilution 1:200, CST), SIRT6 (dilution 1:400, Abcam, Cambridge, UK), and HDAC2 (dilution 1:200, CST) at 4 °C. After washing with PBS, the sections were incubated with a peroxide-conjugated secondary antibody at room temperature for 1 h. Observations were made under an optical microscope (Olympus, BX43, Tokyo, Japan).

### 2.5. Protein Extraction and Trypsin Digestion

Following the ABX procedure, the mice were humanely euthanized, and their ceca were collected. The CON group consisted of three mice, while another set of three mice was designated for the ABX group, with each group undergoing three replicates. Notably, the ceca were promptly isolated and frozen using liquid nitrogen to prevent degradation. To eliminate any potential impact from circadian rhythms, sample collection was performed concurrently, ensuring that all specimens were gathered simultaneously. The samples were subsequently stored at −80 °C until further analysis. The cellular powder was then treated with a lysis buffer and a protease inhibitor, followed by sonication with a high-intensity ultrasonic processor (Scientz, Ningbo, China). After centrifugation at 12,000× *g* for 10 min at 4 °C to remove debris, the supernatant, which contained the protein solution, was collected and quantified for protein concentration utilizing a BCA kit. The protein solution was subjected to treatment with 5 mM dithiothreitol at 56 °C, then alkylated using 11 mM iodoacetamide at 25 °C in a light-protected environment. To reduce the urea concentration to below 2 M, 100 mM TEAB was incorporated to dilute the protein sample. For the initial overnight digestion, trypsin was used at a 1:50 trypsin-to-protein mass ratio to commence the digestion process, and the resulting peptides were desalted employing a C18 solid-phase extraction column. The procedure for protein extraction and breakdown was consistent for both the proteome and the acetylome.

### 2.6. Acetylated Peptide Enrichment and High-Performance Liquid Chromatography (HPLC) Separation and Mass Spectrometry (MS)

The HPLC-MS analysis was conducted following our previous protocol [23]. In this procedure, peptides were first solubilized in an immunoprecipitation buffer consisting of 100 mM NaCl, 1 mM EDTA, 50 mM Tris-HCl, and 0.5% NP-40, adjusted to a pH of 8.0. The resulting supernatant was then transferred into pre-washed antibody resin (PTM104, Jingjie, Hangzhou, China) and allowed to stir overnight at a temperature of 4 °C. Subsequent to washing the resin with both the immunoprecipitation buffer and deionized water, the peptides that had adhered to the resin were eluted using 0.1% trifluoroacetic acid and subsequently freeze-dried. For desalting, C18 ZipTips micro-columns were utilized. The peptides were then dissolved in a mobile phase A comprising 0.1% formic acid and 2% acetonitrile, followed by separation on an UltiMate 1000 ultra-high-performance liquid chromatography system manufactured by Thermo Fisher in Waltham, MA, USA. Mobile phase B consisted of acetonitrile that contained 0.1% formic acid. The liquid chromatography gradient was set as follows: during the initial 0 to 18 min, B ranged from 9% to 24%; from min 18 to 22, it escalated from 24% to 35%; from min 22 to 26, it increased from 35% to 90%; and from min 26 to 30, B remained at 90%, maintaining a flow rate of 450 nl/min. Ionization of the peptides was achieved using a capillary ion source operating at 1.6 kV. Data acquisition was carried out with the timsTOF Pro MS (Bruker, Billerica, MA, USA) in a data-independent parallel accumulation serial fragmentation (dia-PASEF) mode. The mass spectrometry scan range during the first stage was from 100 to 1700 *m*/*z*, followed by eight PASEF mode acquisitions for each initial stage of mass spectrometry data collection. For the second stage of mass spectrometry, the scan range was set between 425 and 1025 *m*/*z* with a 25 *m*/*z* window. Processing of the raw data was executed using Spectronaut software (v. 17.0), with protein identification conducted against the Mus_musculus_10090_SP_20230103.fasta database. Protein domains were detected and described by utilizing the Pfam database alongside the PfamScan v.1.6 tool. For annotating the subcellular localization of proteins, the WolF Psort software (version 1.0) was employed. We used the protein lysine modification database (version 3.0) to investigate the relationships among PTMs in our dataset, identify novel acetylation sites, and examine overlaps with different types of PTMs. The intensity values from all samples were assessed through the Pearson correlation coefficient (PCC) method. The analysis was conducted with fold change thresholds set at either >1.5 or <0.667, combined with a *p*-value < 0.05, which was then used for further analyses.

### 2.7. Statistical Analysis

All experiments were performed in triplicate, and data were presented as mean ± standard deviation. Statistical analyses were performed with GraphPad Prism (v. 10.1.2). Group differences were assessed using Student’s *t*-test and Fisher’s exact test (for two groups), * *p* < 0.05, ** *p* < 0.01, *** *p* < 0.001.

## 3. Results

### 3.1. Gut Microbiota Dysbiosis in Mice Leads to Fluctuations in the Levels of Acetyltransferases and Deacetylases

**Figure 1:**
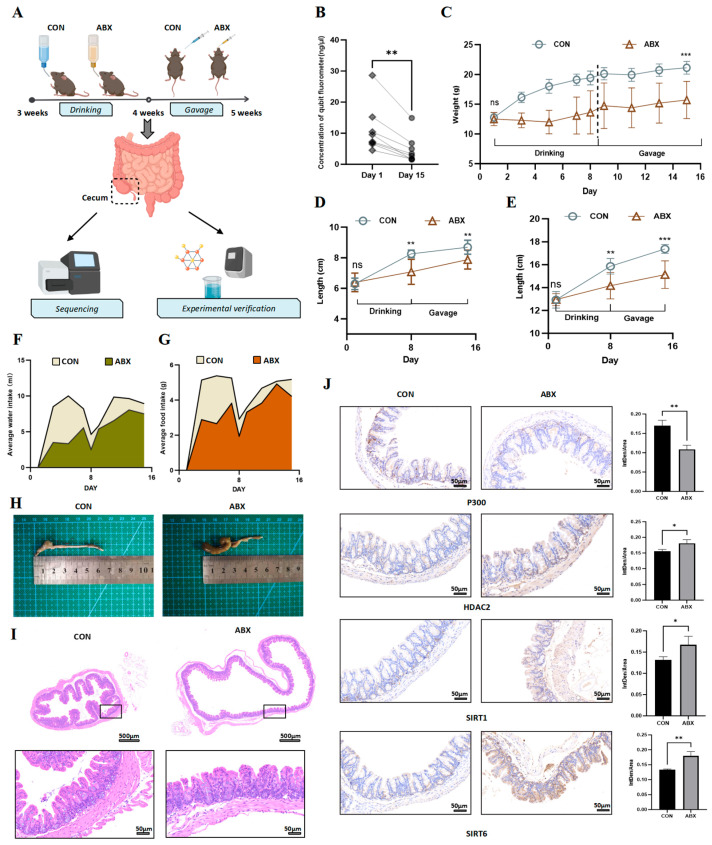
Establishment of ABX-induced intestinal microbiota dysbiosis model in mice and response from acetyltransferases and deacetylases. (**A**) The schematic diagram illustrates the process of constructing a model of ABX-induced gut microbiota dysbiosis in mice, along with the corresponding analysis plan. (**B**) Changes in bacterial DNA concentration in the feces of the ABX group before and after modeling. (**C**) Weight changes of the two groups during modeling. Weight was measured every 2 days starting from first day. Before and after switching from water replacement to gavage intervention, weight was measured daily. (**D**) Changes in the length from the nose tip to the genitalia of the two groups, measured weekly. (**E**) Changes in the length from the nose tip to the tail tip of the two groups, measured weekly. (**F**) Water intake during modeling, compared as the average per mouse. (**G**) Food intake during modeling, compared as the average per mouse. (**H**) Photos and (**I**) HE staining showing morphological or structural abnormalities of the cecum under ABX. (**J**) IHC staining was used to detect the protein expression of P300, HDAC2, SIRT1, and SIRT6. (Data are mean ± SD, *n* ≥ 3). Group differences were assessed using Student’s t-test (for two groups), * *p* < 0.05, ** *p* < 0.01, *** *p* < 0.001 and ns, no significant.

To investigate the alterations in lysine acetylation abundance resulting from early-life gut dysbiosis in mice, we induced gut dysbiosis in postweaning mice through ABX. Given the young age of the subjects, we permitted them to freely consume water containing a four-antibiotic cocktail during the first week. During the following week, we administered this four-antibiotic cocktail daily via gavage. Two weeks later, cecal samples were collected from the mice, and Figure 1A illustrates our modeling process. Additionally, fecal samples were obtained both prior to and following intervention to assess the bacterial DNA concentration. A significant decrease in the bacterial DNA concentration was observed in feces after ABX treatment, indicating a substantial reduction in the gut microbiota (Figure 1B). Throughout our modeling study, statistical evaluations were conducted on various parameters obtained from the mice, including body weight, body length, water consumption, and food intake. Compared with control mice, ABX-treated mice presented a markedly reduced rate of weight gain (Figure 1C). We measured the lengths from the nose tip to the genitalia as well as from the nose tip to the tail tip; the findings reveal that growth in length in the ABX group was less pronounced than that in the control (CON) group (Figure 1D,E). This observation suggested an association between disruption in the gut microbiota due to ABX treatment and the overall growth and development of these mice. Furthermore, both water and food consumption levels within the ABX group were significantly lower than those recorded for the CON group (Figure 1F,G), with particularly notable discrepancies found during the initial week relative to observations made during the second week. Macroscopic and pathological examinations of mice cecum were also performed. Upon dissection, the cecum from ABX-treated mice appeared significantly enlarged and congested compared with those from control subjects (Figure 1H). From a pathological standpoint, following the administration of ABX, we observed significant structural abnormalities in the cecum, characterized by discontinuity in the mucosal epithelium and deformation of the crypt architecture. These findings indicate that the intestinal mucosal barrier was compromised (Figure 1I). Furthermore, we conducted IHC analyses of acetyltransferase P300 and the deacetylases HDAC2, SIRT1, and SIRT6. Our results reveal that ABX treatment significantly downregulated P300 as well as the deacetylases HDAC2, SIRT1, and SIRT6 (Figure 1J). These observations suggest that dysbiosis of the gut microbiota during early life can impede growth and development in mice. Additionally, alterations in gut microbiota composition affect the expression levels of both acetyltransferases and deacetylases.

### 3.2. Gut Microbiota Dysbiosis in the Intestinal Microbiota of Mice Leads to Changes in the Cecal Proteome

**Figure 2:**
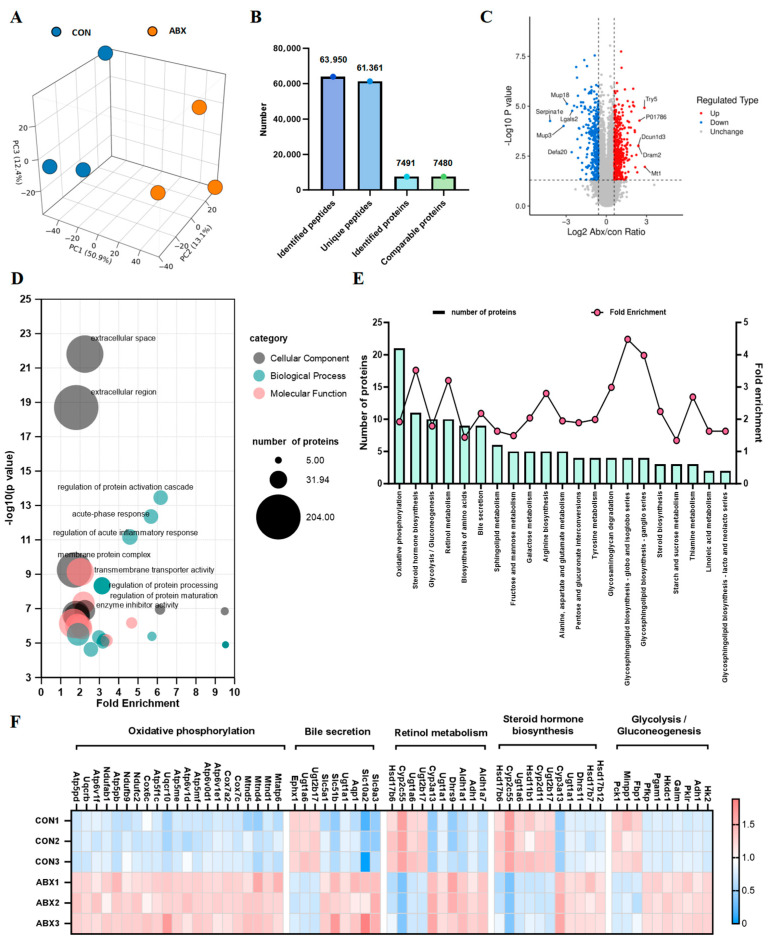
Proteomic analysis of mouse cecum (**A**) PCA plot of two sample groups in the proteomics dataset. (**B**) The number of detected peptides and proteins in the samples. (**C**) The volcano plot illustrates the differential changes in proteins (fold change > 1.5 or <0.667, with *p* value < 0.5). (**D**) The bubble chart illustrates the GO analysis of proteomics, highlighting the top 10 entries by *p* value. (**E**) The bar-line chart summarizes the KEGG pathway enrichment analysis related to metabolism in proteomics. (**F**) The heatmap displays the differentially expressed proteins involved in oxidative phosphorylation, steroid hormone biosynthesis, glycolysis/gluconeogenesis, retinol metabolism, and bile secretion.

Despite the observed dysbiosis of the gut microbiota in mice exposed to ABX, the specific mechanisms underlying the disturbance of the proteome and acetylome in the cecal tissue remain unclear. To fill this gap, we utilized mass spectrometry to examine variations in protein levels and acetylation in mice. We evaluated alterations in protein expression within the cecal tissue of mice that received ABX treatment using proteomic methods. First, we analyzed the proteome of the cecal tissue of the mice. Principal component analysis (PCA) revealed a significant separation between the CON and ABX groups, clearly distinguishing the differences between the two groups (Figure 2A). We identified 63,950 peptide sequences in the cecal tissues of mice from the ABX and CON groups. From these, we identified 7491 proteins, of which 7480 were quantified using proteomic profiling (Figure 2B). A total of 793 differentially expressed proteins (DEPs) were identified, comprising 453 that were upregulated and 340 that were downregulated (Table S1). The volcano plot depicts the alterations in the differentially expressed proteins (Figure 2C). We observed significant upregulation of SIRT1 (Figure S1A). Additionally, we explored the functional enrichment associated with the proteins that were differentially expressed in the cecum of mice subjected to ABX. The bubble plot presents the entries from the GO analysis across three levels: biological process (BP), molecular function (MF), and cellular component (CC), highlighting the top 10 entries. The BP was enriched in the regulation of protein activation cascades, regulation of acute inflammatory responses, and regulation of protein processing. The CC was enriched in the extracellular space, extracellular region, and membrane protein complex. MF entries were enriched in transmembrane transporter activity and enzyme inhibitor activity (Figure 2D, Table S2). In the KEGG pathway enrichment analysis, we found that the most enriched entries involved metabolic pathways, with a total of 128 enriched proteins. We summarized the changes in differentially expressed proteins (DEPs) related to metabolic pathways (Figure S2B, Table S3). Further observation revealed the five metabolic pathways with the greatest number of DEPs, including oxidative phosphorylation, steroid hormone biosynthesis, glycolysis/gluconeogenesis, retinol metabolism, and bile secretion (Figure 2E). Additionally, we present the DEPs of these metabolic pathways in a heatmap (Figure 2F). Furthermore, we identified the subcellular distribution of DEPs following ABX intervention. These proteins were primarily located in the cytoplasm (25.6%), extracellular space (22.5%), and plasma membrane (16.3%) (Figure S1C, Table S4). These findings indicate that gut microbiota dysbiosis can lead to differential protein changes in various cellular components of the cecal tissue in mice during early life.

### 3.3. Identification of Lysine-Acetylated Proteins and Sites in the Cecal Tissue of Mice Induced by Gut Microbiota Dysbiosis

**Figure 3:**
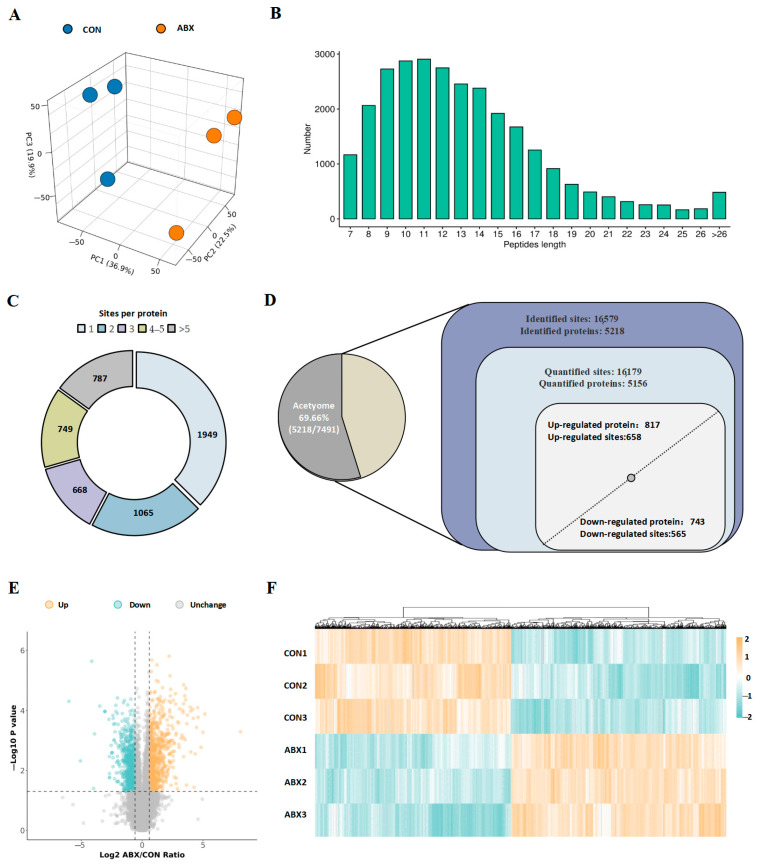
Characteristic motif analysis of acetylation sites in mice cecum. (**A**) PCA distinguished the control and ABX groups by their unique signatures. (**B**) Distribution of peptide lengths for all identified acetylated peptides. (**C**) Pie chart of acetylation sites per modified protein. (**D**) Identified acetylated proteins and sites are shown. Venn diagrams display acetylation sites (with corresponding proteins in brackets). Proteins with fold changes >1.5 or <0.667 and *p* < 0.05 were considered significantly upregulated or downregulated. (**E**) Volcano plot and (**F**) hierarchical clustering heatmap of differential Kac sites.

In the cecal tissues of the mice, we observed intricate changes in acetylated proteins and their respective sites. Similarly, we initially assessed the quality of the mass spectrometry (MS) data to ensure their reliability. We employed stringent filtering criteria, specifically establishing a significance threshold of *p* < 0.05 and a minimum fold change threshold of 1.5, to refine our list of potentially relevant alterations in lysine acetylation levels. We identified differences between the CON group and the ABX group using PCA, which revealed significant differences between the two sample sets (Figure 3A). Our evaluation indicated that all acetylated peptide lengths ranged from 7 to 47 amino acids, with the majority falling within the range of 7 to 20 amino acids, accounting for 92.7% of the total count. The length distribution of the identified peptides met established quality control standards (Figure 3B). Among these acetylated proteins, 1950 (37.4%) possessed a single Kac site, whereas 787 (15.1%) presented more than five Kac sites (Figure 3C). Furthermore, we analyzed proteins with over twenty acetylation sites. This subset included 24 proteins, such as Flna (54 sites), Plec (49 sites), and Dync1h1 (46 sites), among others (Table S5). In total, we identified an impressive array of 16,579 acetylation sites distributed across 5218 proteins. These findings suggest that approximately 69.66% of modified proteins exhibited evidence of acetylation (Figure 3D, left). Among the 16,179 acetylation sites quantified across 5156 proteins, the number of upregulated differentially acetylated proteins (DAPs) and differentially acetylated sites (DASs) slightly surpassed that of downregulated proteins (Figure 3D, right). The volcano plot illustrates the variations in differentially acetylated proteins, with simultaneous labeling of the names of the top five upregulated and downregulated differential proteins (Figure 3E, Table S6). The heatmap reveals a significant alteration in the overall acetylation profile within the cecum induced by ABX (Figure 3F). In summary, these findings indicate that dysbiosis of the gut microbiota during early life can substantially disrupt protein acetylation levels.

### 3.4. Characteristics of Acetylation Sites in Mouse Cecal Tissue Induced by Gut Microbiota Dysbiosis

**Figure 4:**
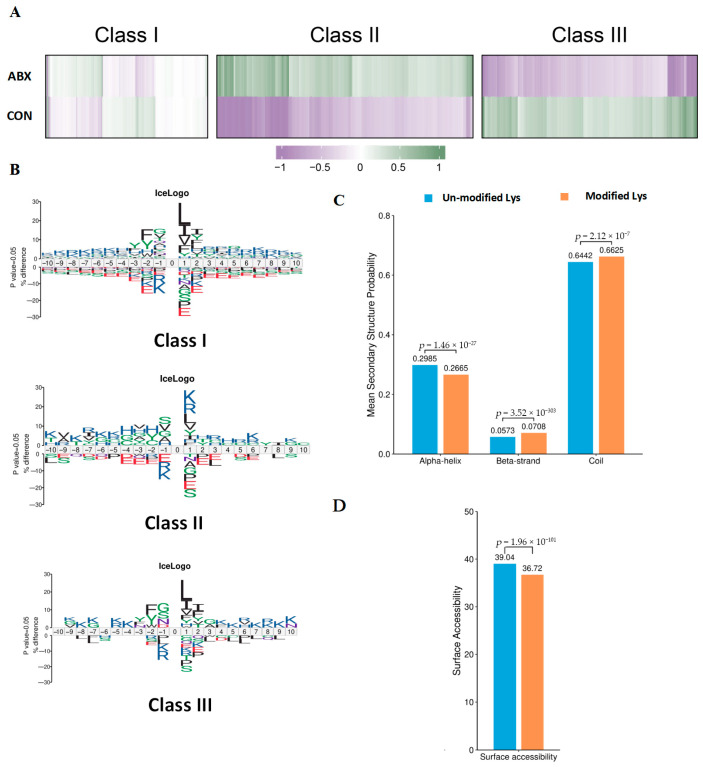
Characteristics of acetyl isoforms in response to gut microbiota dysbiosis. (**A**) Based on the overall trends of acetylation sites under ABX and control conditions, these sites were categorized into three classes. Class I sites exhibited no significant change, Class II sites were upregulated, and Class III sites were downregulated. (**B**) Predicted amino acid motifs for each group were identified using a binomial test (*p* < 0.05). (**C**) The secondary structure of proteins and (**D**) the prediction of protein surface accessibility for all differentially acetylated sites were assessed by the Wilcoxon rank sum test.

To explore the distribution of acetylated sites identified following ABX treatment, we categorized all the acetylated sites into three groups: Class I included unregulated sites, Class II mainly comprised sites that were upregulated due to ABX, and Class III predominantly featured downregulated sites (Figure 4A). The emphasis on particular amino acids arises from their important functions within the sequence context surrounding the modification sites. Acetylation predominantly occurs on lysine (K) residues. The amino acids adjacent to the modification sites can affect the occurrence, stability, and functionality of these modifications. Motif analysis was conducted for every group to gain insight into their physical traits. Our results demonstrate that there was a tendency for Class II sites to favor aspartic acid (D) at the −1 position, in contrast to Class I sites, which showed a preference for glycine (G) in the same position. Furthermore, we observed that Class III sites showed a preference for isoleucine (I) at the +1 position, whereas Class I sites favored leucine (L) at that position (Figure 4B). Compared with lysine residues that were not modified, DASs were markedly enriched in beta strands (*p* = 3.52 × 10^−303^) and coils (*p* = 2.12 × 10^−7^), although there was a decrease in enrichment within alpha helices (*p* = 1.46 × 10^−27^) (Figure 4C). Similarly, DASs displayed decreased accessibility with respect to surface exposure (*p* = 1.96 × 10^−101^) (Figure 4D). These findings imply that DASs induced by ABX in the cecum could alter protein function by modifying the preferences for adjacent amino acids, beta-strand secondary structures, and surface exposure.

### 3.5. Analysis of the Subcellular Distribution and Gene Ontology of Acetylated Proteins That Are Significantly up- and Downregulated in Gut Microbiota Dysbiosis

**Figure 5:**
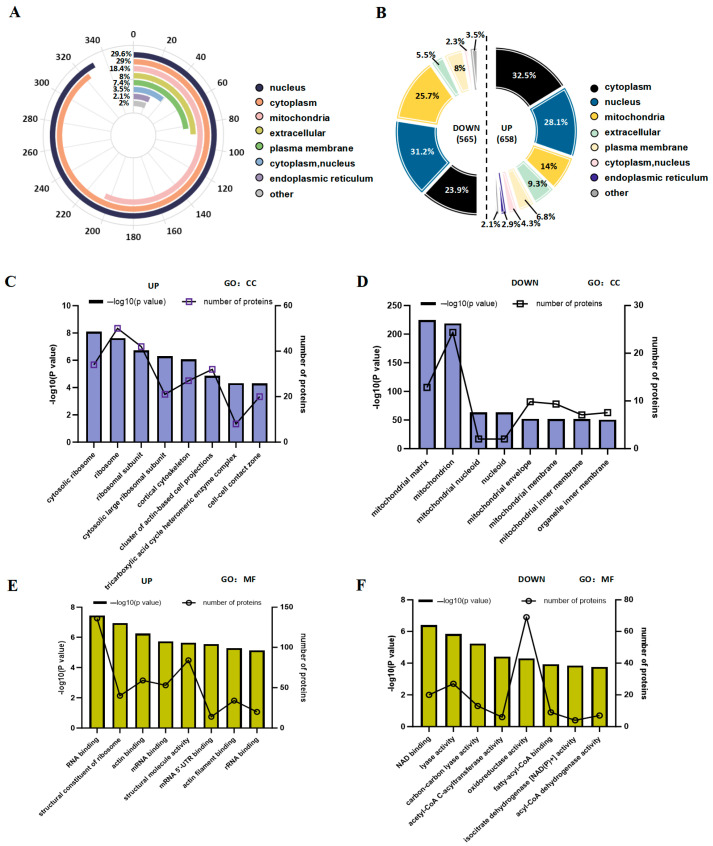
Subcellular distribution and GO analysis of acetylated proteins. (**A**) The distribution ratio of differential acetylation sites in subcellular compartments. (**B**) The pie chart illustrates the distribution of upregulated and downregulated differential acetylation sites within subcellular compartments. (**C**) Enrichment of upregulated differential acetylation sites in the CC of GO analysis. (**D**) Enrichment of downregulated differential acetylation sites in the CC of GO analysis. (**E**) Enrichment of upregulated differential acetylation sites in the MF of GO analysis. (**F**) Enrichment of downregulated differential acetylation sites in the MF of GO analysis.

Additionally, we investigated the subcellular localization of DAPs following ABX intervention. The majority of these proteins were identified in the nucleus (29.6%), cytoplasm (29%), and mitochondria (18.4%) (Figure 5A). Analyzing both upregulated and downregulated DAPs provided insights into their specific subcellular distributions. Among the upregulated DAPs, the predominant subcellular locations included the cytoplasm (32.5%), nucleus (28.1%), and mitochondria (14%). Conversely, for the downregulated DAPs, the three leading subcellular locations were the nucleus (31.2%), mitochondria (25.7%), and cytoplasm (23.9%), as illustrated in Figure 5B. We demonstrated that notable differences existed between the upregulation and downregulation of various acetylated proteins with respect to their subcellular localization. Furthermore, to explore the biological functions and networks associated with distinct acetylated lysine residues, we performed GO analysis. In terms of cellular component categories, the top ten enriched terms for upregulated acetylated proteins included cytosolic ribosome, ribosome, ribosomal subunit, cytosolic large ribosomal subunit, cortical cytoskeleton, cluster of actin-based cell projections, tricarboxylic acid cycle heteromeric enzyme complex, and cell-cell contact zone, as shown in Figure 5C and Table S7. The entries related to downregulated acetylated protein enrichment included the mitochondrial matrix, mitochondrion, mitochondrial nucleoid, nucleoid, mitochondrial envelope, mitochondrial membrane, mitochondrial inner membrane, organelle inner components, and membranes (Figure 5D; Table S7). Analysis of molecular function (MF) revealed that the top 10 upregulated acetylated proteins were significantly enriched in RNA-binding activities, structural constituents of ribosomes, actin-binding capabilities, mRNA-binding interactions, structural molecule activity, mRNA 5’-UTR-binding properties, actin filament-binding functions, and rRNA-binding activities (Figure 5E; Table S7). In contrast, the top 10 downregulated acetylated proteins were enriched in NAD-binding activities as well as various enzymatic functions, including lyase activity—specifically, carbon-carbon lyase activity—and acetyl-CoA C-acyltransferase activity oxidoreductase activity. Additionally, fatty-acyl-CoA-CoA-binding capacity and isocitrate dehydrogenase [NAD(P)+] activity alongside acyl-CoA dehydrogenase functionality were noted (Figure 5F, Table S7). These findings indicate that the alterations in acetylation induced by antibiotic treatment primarily involve distinct molecular functions encompassing processing and cellular components within cytoplasmic ribosomes and mitochondria.

### 3.6. Enrichment Clustering of Protein Domains and Biological Function Analysis of the Kac Proteome in Gut Microbiota Dysbiosis

**Figure 6:**
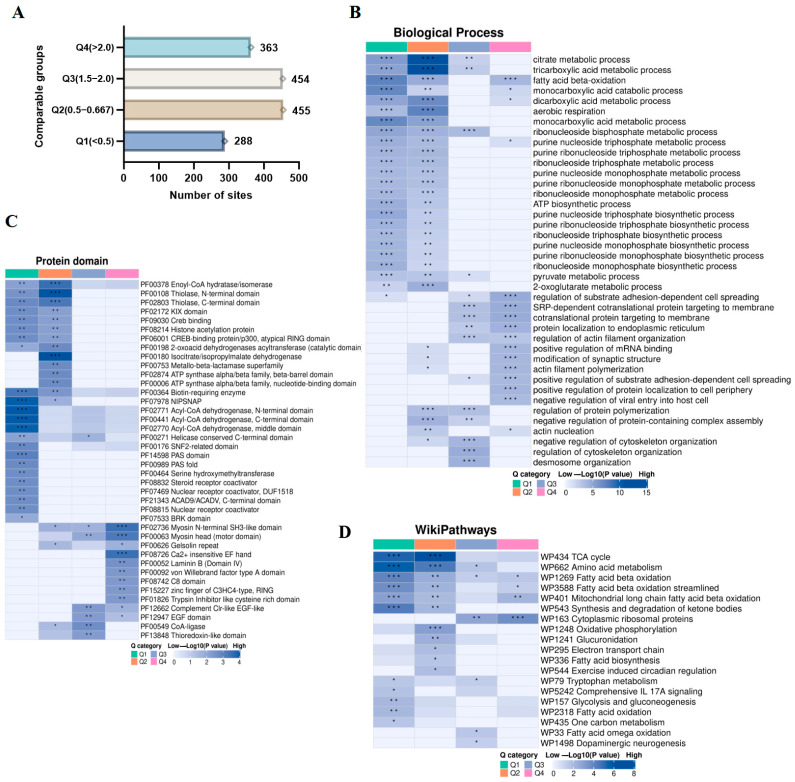
Functional analysis of acetylated proteins with foldchange. (**A**) Differential acetylation sites were categorized into four groups based on fold change. (**B**) The BP in the GO analysis was conducted for acetylated proteins with fold changes (Q1–Q4). (**C**) The heatmap illustrates the predominant protein domains enriched by the acetylated proteins. (**D**) WikiPathways analysis. Group differences were assessed using Fisher’s exact test. * *p* < 0.05, ** *p* < 0.01, *** *p* < 0.001.

We subsequently categorized the acetylated sites that exhibited significant changes into four distinct groups (Q1–Q4) on the basis of their fold change (Figure 6A). GO analysis of the BP indicated that Q1 was associated primarily with the breakdown of fatty acids via beta-oxidation, as well as the catabolism and metabolism of monocarboxylic acids. Q2 was largely linked to metabolic processes involving tricarboxylic acids, citrate metabolism, and aerobic respiration. Q3 focused on the regulation of cytoskeleton organization, desmosome organization, and negative regulation of cytoskeleton organization. In contrast, Q4 emphasized the regulation of substrate adhesion-dependent cell spreading, SRP-dependent cotranslational protein targeting to membranes, and cotranslational protein targeting to membranes (Figure 6B; Table S8). Protein domain analysis revealed that Kac proteins in Q1 were predominantly involved in both the N- and C-terminal domains of acyl-CoA dehydrogenase. Kac proteins within Q2 were chiefly associated with isocitrate/isopropylmalate dehydrogenase and the N-terminal domain of thiolase. In Q3, Kac proteins were related mainly to CoA ligases and possessed a complement Clr-like EGF-like domain. Moreover, the proteins in Q4 were significantly associated with calcium-independent EF-hand motifs and myosin N-terminal SH3-like domains (Figure 6C; Table S9). WikiPathways analysis revealed that Q1 Kac proteins were predominantly associated with amino acid metabolism and the TCA cycle. In contrast, Q2 Kac proteins were linked to both the TCA cycle and amino acid metabolism. Q3 Kac proteins focused primarily on calcium regulation in cytoplasmic ribosomal proteins, alongside their involvement in amino acid metabolism. Moreover, Q4 Kac proteins participated in the functions of cytoplasmic ribosomal proteins and the β-oxidation of long-chain fatty acids within mitochondria (Figure 6D, Table S10). These findings suggest that gut microbiota influences multiple biological processes and protein domains by altering the acetylation levels of relevant proteins in the cecum of mice treated with ABX.

### 3.7. The Impact of Acetylated Lysine on Gut Microbiota Dysbiosis During Early Life Can Disrupt Metabolic Processes

**Figure 7:**
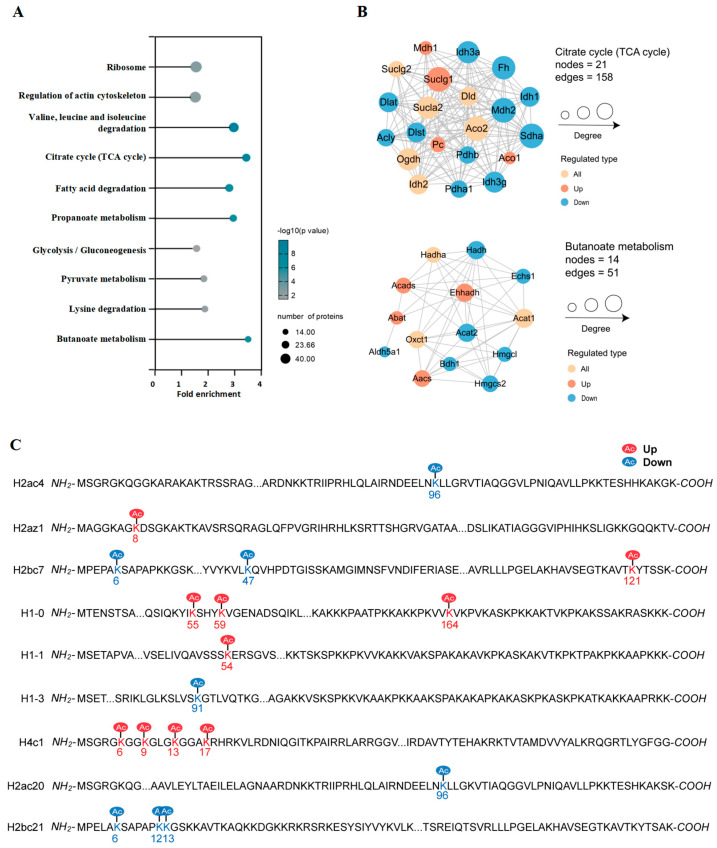
KEGG pathway enrichment analysis of acetylated proteins and histone acetylation site in mice cecum. (**A**) Pathway enrichment analysis of significantly enriched differentially acetylated proteins. (**B**) Focused PPI on the differential acetylation of citrate cycle (TCA cycle) and butanoate metabolism. (**C**) Identification and quantitative analysis of histone Kac sites between the CON group and the ABX group. Histone lysine sites with varying levels of Kac are color-coded.

We also analyzed the changes in KEGG signaling pathways induced by gut microbiota dysbiosis. In Figure 7A, we present the top 10 pathways. Notably, the citrate cycle (TCA cycle) and butanoate metabolism pathways were significantly enriched. Studying protein-protein interactions (PPIs) is a key step in revealing protein functions and aids in the investigation and manipulation of critical cellular processes. To better understand the interactions among the DAPs, we conducted a PPI network analysis on the basis of the characteristic that highly clustered proteins typically have similar or related functions. We focused on highly confident DAPs related to the citrate cycle (TCA cycle) and butanoate metabolism, detailing the connections among their associated DAPs (Figure 7B). The citrate cycle (TCA cycle) cluster included 11 DAPs with downregulated acetylation sites and 4 DAPs with upregulated acetylation sites (Figure S2A,B). The acetylation at Mdh2 K301/K329 and Idh1 K159 was reduced. It has been reported that the acetylation modifications at K301 and K329 on Mdh2 can occur, with K329 linked to a higher apparent affinity for malate and a reduced turnover rate [24]. Idh1 catalyzes the interconversion of isocitrate and α-ketoglutarate in the cytoplasm through NADP/NADPH, participating in the metabolism of carbohydrates, lipids, and amino acids. Previous studies have shown that the acetylation modification of Idh1 is significantly associated with energy metabolism [25]. Additionally, 6 DAPs presented both upregulated and downregulated acetylation sites, namely, Suclg2, Dld, Sucla2, Aco2, Ogdh, and Idh2, among which the acetylation levels of most sites were reduced (Figure S2C). In the butanoate metabolism cluster, there were seven downregulated DAPs with acetylation sites and four upregulated DAPs (Figure S2D,E). ACADS has been noted to undergo increased acetylation modifications at K343. The lysine acetylation of ACADS may alter its conformation, regulating its catalytic efficiency towards SCFAs, which in turn affects ATP production [26]. The acetylation at the K101 site of Echs1 decreased. ECHS1 is another key enzyme that facilitates the conversion of butyrate into acetyl-CoA [27]. Additionally, there were three DAPs with both upregulated and downregulated acetylation sites, namely, Hadha, Acat1, and Oxct1. We also observed that more than half of the acetylation sites in these DAPs presented decreased acetylation levels (Figure S2F). In the TCA cycle cluster, the acetylation levels of Idh3a, Mdh2, and Pdha1 were reduced, whereas their protein levels remained unchanged. These genes encode key enzymes in the TCA cycle, which are translocated from the mitochondria to the nucleus during cellular reprogramming, increasing the levels of acetyl-CoA and promoting histone acetylation, significantly affecting the expression of pluripotency-related genes and the chromatin structure [28]. The key role of histone modifications lies in the epigenetic regulation of gene “switch” states, thereby influencing changes in chromatin structure and function, cell differentiation and development, and the occurrence of diseases [29]. We demonstrated the acetylation status of classical histones and their variants. A total of 18 acetylated lysine residues on core histones were identified and quantified, among which 10 residues exhibited relatively high acetylation levels in the cecal tissue of ABX group mice, whereas the acetylation levels of the remaining 8 residues were reduced (Figure 7C, Table S11). In summary, these results further support the notion that the complex changes induced by dysbiosis of the gut microbiota require the synergistic action of various cellular components. These findings indicate that the pivotal regulatory role of Kac may exert complex and crucial regulatory effects on host metabolism during early dysbiosis of the gut microbiota.

## 4. Discussion

From the earliest stages of life, newborns establish a close interactive relationship with their gut microbiome. During this critical developmental phase, significant advancements and refinements in their metabolic systems, immune networks, digestive functions, and neurocognitive mechanisms occur [30]. The gut microbiota influences host cell functions through its metabolites; alterations in its composition can affect gene expression in the host via complex epigenetic regulation, which may even impact the physiological and pathological states of the host [31]. SCFAs produced by various bacteria such as anaerobic bacilli and bifidobacteria are considered unique epigenetic regulators that link diet, metabolism, and gene expression by depositing distinct acyl marks on histones, thereby influencing chromatin accessibility and gene expression [32,33]. Indole-3-lactic acid is primarily produced by probiotics such as lactobacillus and bifidobacterium. It regulates chromatin accessibility and modulates cholesterol metabolism, impacting the epigenetic mechanisms underlying the occurrence and development of colorectal cancer [34,35]. Gut microbiota also produces various vitamins, such as folate (vitamin B9) synthesized through one-carbon metabolism by bifidobacteria and lactobacilli, which promotes DNA methylation [8]. A key mechanism for regulating interactions between the gut microbiota and the host is the acetylation of lysine residues. For example, butyrate, a prominent metabolite produced by the gut microbiota, can inhibit the differentiation of intestinal stem cells into tuft cells by acting on HDAC3 and its target genes [36]. However, the precise mechanisms by which the gut microbiota regulates host states via global lysine acetylation during early life stages remain unclear.

In this study, we developed a mouse model of antibiotic-induced gut microbiota dysbiosis using weaned mice (3 weeks old) to simulate the early life stages of children. A comprehensive assessment of acetylation in cecal tissue was performed to investigate the global changes in acetylation resulting from gut microbiota dysbiosis. Our findings revealed that numerous proteins are involved in metabolic pathways such as oxidative phosphorylation, steroid hormone biosynthesis, glycolysis/gluconeogenesis, retinol metabolism, and bile secretion (Figure 2E). We identified 16,579 acetylation sites across 5218 proteins. Further analysis revealed that among the 5156 quantified proteins and 16,179 acetylation sites, 1223 acetylation sites on 1560 proteins exhibited differential regulation under antibiotic exposure, accounting for 69.66% of the proteome within the entire cecal tissue (Figure 3D). A significant number of acetylation sites affected by gut microbiota dysbiosis were located near basic amino acids and may influence the secondary structure of proteins (Figure 4). These findings may suggest that the gut microbiota serves as a major regulator of protein acetylation in the cecal tissue of mice during early life. Furthermore, lysine acetylation plays a critical regulatory role in various metabolic processes, including the citrate cycle (TCA cycle), butanoate metabolism, pyruvate metabolism, glycolysis/gluconeogenesis, and fatty acid biosynthesis (Figure 7A). Additionally, we conducted preliminary investigations into alterations in histone acetylation induced by gut microbiota dysbiosis (Figure 7C). Therefore, we hypothesize that global lysine acetylation could play an essential role in regulating host metabolic pathways influenced by the gut microbiota during early development.

Both glycolysis and gluconeogenesis were significantly enriched in acetylation and protein modifications (Figure 2E and Figure 7A). Previous studies have systematically characterized human gut microbiome samples utilizing mass spectrometry-based macromodification proteomics. These findings indicate that lysine acetylation is prevalent within the human gut microbiome and is particularly associated with the glycolytic metabolic pathway and SCFA production [21]. Under dietary restrictions, the gut microbiota can inhibit lymphocyte generation by downregulating glycolysis [37]. Furthermore, the gut microbiome modulates the glycolytic metabolism of porcine intestinal epithelial cells through lysine succinylation modifications [38]. Our investigation revealed that early-life dysbiosis of the gut microbiota in mice leads to alterations in proteins and acetylated proteins related to glycolysis and gluconeogenesis. These findings underscore the critical role of the gut microbiota in host metabolism linked to glycolysis via lysine acetylation. Our proteomic analysis revealed notable enrichment of proteins involved in various metabolic pathways, such as oxidative phosphorylation, steroid hormone biosynthesis, retinol metabolism, and bile secretion (Figure 2F). These metabolic processes are regulated by the gut microbiota and play a role in host physiological changes through epigenetic regulation [9,39,40,41]. Our results suggest that dysbiosis of the gut microbiota in early life can affect host metabolic changes through mechanisms partly involving lysine acetylation.

Our findings indicate that dysbiosis of the gut microbiota significantly alters the acetylome profile in the cecum, which may play a crucial role in modifying the acetylation of the tricarboxylic acid (TCA) cycle (Figure 7A,B). The TCA cycle is an essential metabolic pathway within cells that is responsible for generating energy and various intermediate metabolites. Changes in the gut environment can suppress the growth of certain microbial communities while allowing others that utilize intermediates from the TCA cycle to proliferate excessively, resulting in an imbalance in microbial composition [42]. We observed hypoacetylation at K159 of IDH1 (Figure S2B). Previous studies have demonstrated that the HDAC inhibitor ACY1215 enhances metabolism by influencing IDH1 at K93 [25]. In intestinal tumors, TNF-α disrupts interactions between IDH1 and the deacetylase SIRT1, leading to decreased IDH1 protein stability. Conversely, the absence of TNF-α can restore low acetylation levels at K115 on IDH1, resulting in the accumulation of reductive metabolites within tumor cells [43]. MDH2 serves as a pivotal molecule maintaining energy metabolism homeostasis within the mitochondrial TCA cycle (Figure S2B). Research has shown that MDH2 interacts with its upstream and downstream partners—FH and CS—to form metabolic compartments. This interaction facilitates the ability of MDH2 to complete reactions characterized by reverse standard Gibbs free energy (ΔG0’) through substrate chaining. Mutations occurring at positions K185, K301, K307, and K314 within MDH2 compromise the stability of these metabolic compartments, ultimately leading to mitochondrial metabolic abnormalities [44]. MDH2 acetylation not only influences its own enzymatic activity but also impacts its cellular metabolism through interactions with other metabolic enzymes. Mutations at the acetylation sites K159, K185, and K404 of MDH2 can disrupt its binding affinity with glutamate oxaloacetate transaminase 2 (GOT2), thereby affecting the metabolic flux within the cell [45]. We observed significant alterations at K301 and K329 of MDH2, with K301 being identified as the primary site for acetylation in MDH2 [46]. However, its role in regulating the gut microbiota through acetylation remains unclear. Our study offers new insights into the potential mechanisms by which acetylation may contribute to gut microbiota dysbiosis during early childhood and suggests that mitochondrial metabolism regulated by the TCA cycle could play a critical role in maintaining children’s health during this formative period.

Our analysis revealed a significant enrichment in butanoate metabolism (Figure 7A,B). Butyrate is one of the key SCFAs present in the intestine and enhances intestinal barrier function by modulating the expression of the tight junction protein claudin-1 and mucin proteins [47]. It enters epithelial cells via specific transport proteins and subsequently diffuses into the mitochondria, where it undergoes β-oxidation to produce acetyl-CoA. This acetyl-CoA then serves as a substrate for both the TCA cycle and the electron transport chain, facilitating ATP production [48]. We observed ACADS acetylation at K343 (Figure S2D). SCFAs are metabolized into acetyl-CoA through β-oxidation, which is essential for fueling the TCA cycle. ACADS plays a pivotal role in this metabolic pathway by catalyzing the oxidation of short-chain acyl-CoA to generate acetyl-CoA, thereby providing the substrates necessary for TCA cycle activity [49]. In colonic crypts, ACADS activity is critical for maintaining normal intestinal stem cell function. Research has demonstrated that mice deficient in ACADS exhibit reduced proliferation capacity among intestinal stem cells [50]. We observed low K101 acetylation levels in ECHS1 (Figure S2E). Previous studies have indicated that branched-chain amino acids (BCAAs) in colorectal cancer enhance the acetylation of K204 in ECHS1, thereby impairing its ability to bind to enoyl-CoA and diminishing its catalytic activity. This alteration facilitates the accumulation of BCAAs within cells and contributes to cancer progression. ECHS1 acetylation is crucial for regulating cellular metabolism and growth [51]. HADHA serves as a key enzyme in mitochondrial β-oxidation [52]. Acetyl-CoA produced by HADHA directly enters the TCA cycle, generating intermediates such as citrate, α-ketoglutarate, and succinyl-CoA. These intermediates not only participate in energy metabolism but also act as precursors for biosynthetic processes [53]. The K303 residue of HADHA is the primary site for acetylation and is essential for modulating the enzymatic activity of HADHA [54]. Our analysis revealed a decrease in acetylation at residues K295, K406, K411, K414, K415, K644, and K728; conversely, we noted an increase at residues K353 and K540. This discrepancy may reflect the intricate regulatory mechanisms governing this protein within the cellular context (Figure S2F). In macrophages, acetylation at the K303 site of HADHA regulates adaptive responses related to mitochondrial metabolism and morphology, thus influencing inflammatory responses [54]. Currently, there are no reports directly linking HADHA to intestinal function. However, given its role as a pivotal enzyme connecting butanoate metabolism with the TCA cycle, HADHA is likely to play a significant role in regulating host intestinal function.

Evidence suggests that the gut microbiota influences host histone modifications [2]. SCFAs and various metabolites and cofactors, including SAM, acetyl-CoA, ATP, Fe^2+^/Fe^3+^, and α-ketoglutarate, can either inhibit or promote these histone modifications [31]. Research has demonstrated that acetylation, propionylation, and butyrylation occur at the H3K9/27 sites within the intestinal tissues of mice. Inducing dysbiosis in the gut microbiota through antibiotic treatment can lead to alterations in these modifications [14]. Another study revealed a uniform decrease in H4 acetylation levels in the intestines of germ-free female mice, identifying four specific acetylation sites, including H4K4, H4K8, H4K12, and H4K16 [20]. We identified 18 histone acetylation sites that exhibited changes in cecal tissue from mice; among these sites were K5, K8, K12, and K16 on histone H4. These four acetylation sites are commonly associated with gene expression activation [47,48,55]. These findings further underscore the significant role of gut microbiota dysbiosis in modulating the acetylation levels of cecal tissue during early life. These findings suggest that these specific sites play important roles in host epigenetic regulation.

The regulation of acetylation is governed by lysine acetyltransferases (KATs) and lysine deacetylases (KDACs). Our findings demonstrate that exposure to ABX significantly reduces the levels of P300 in the cecum. Early studies have indicated that SCFAs stimulate the activity of the acetyltransferase P300, thereby increasing its capacity and influencing the acetylation status of both histones and nonhistones. The gut microbiota and its metabolites may modulate P300 activity and function through alterations in serine 89 phosphorylation, which subsequently impacts intestinal immune responses, maintains intestinal barrier integrity, and regulates intestinal cell proliferation and differentiation. Furthermore, SIRT1 is markedly upregulated in the cecum of ABX-treated mice. Recent investigations suggest that the gut microbiota can influence host lipid metabolism by regulating the long noncoding RNA Snhg9. In germ-free mouse models, Snhg9 selectively interacts with CCAR2, an inhibitor of the deacetylase SIRT1. This interaction obstructs the binding between CCAR2 and SIRT1, consequently enhancing the enzymatic activity of SIRT1 [56]. This cascade of molecular events ultimately results in impaired intestinal lipid absorption, underscoring the critical role played by microbial-host interactions in metabolic regulation.

We opted to utilize only male juvenile mice primarily to minimize the potential interference of physiological changes induced by the hormonal cycles of female animals on our experimental results, thereby enhancing both the consistency and the comparability of our data. Previous studies have demonstrated that the abundance and diversity of gut microbiota in male fruit flies differ by an order of magnitude compared to females, suggesting that gut microbiota may also have a certain impact on sexual dimorphism [57]. Additionally, alterations in the gut microbiota resulting from ovariectomy can directly lead to metabolic disorders in recipient mice, indicating that the gut microbiota plays a critical mediating role between female hormonal status and metabolic diseases [58]. Furthermore, research has indicated that sex plays a dominant role in shaping the development of gut microbiota during infancy within the first 1–6 months post-birth. The composition of the gut microbiota in female infants may resemble the adult structure earlier than that seen in male infants [59]. This evidence suggests that there are significant differences in gut microbiota attributable to sex. However, we acknowledge that this selection based on sex limits the broader applicability of our findings. In future studies, we will expand our investigation into how sex influences the gut microbiota.

## 5. Conclusions

Our findings suggest that dysbiosis of the gut microbiota during early life can disrupt host metabolism by modulating global protein acetylation. This study investigated the potential mechanisms through which gut microbiota dysbiosis influences host epigenetic modifications, thereby enhancing our understanding of the intricate molecular pathways associated with gastrointestinal diseases in children, as well as their growth and development in early life. The gastrointestinal tract of children demonstrates a markedly greater susceptibility compared to that of adults. Our findings establish a theoretical foundation for the investigation of acetylation-centered interventions, which may contribute to the protection of gut health in pediatric populations suffering from gut microbiota dysbiosis-related diseases.

## Data Availability

The data presented in the study are deposited in the ProteomeXchange repository, accession number PXD064558.

## Supplementary Materials

The following supporting information can be downloaded at: https://www.mdpi.com/article/10.3390/biology14080917/s1, Figure S1: Analysis of DEPs; Figure S2: Histone variants with acetylation. Table S1: DEPs, Table S2: Go analysis of DEPs, Table S3: KEGG of DEPs, Table S4: Subcellular distribution of DEPs, Table S5: Modification site information of lysine acetylation, Table S6: DAPs; Table S7: Go analysis of DAPs; Table S8: GO analysis of DAPs with foldchange, Table S9: DAPs domain; Table S10: DAPs WikiPathway; Table S11: Histone.
