# Supplementary figures and images for: Gut Microbiota Dysbiosis Remodels the Lysine Acetylome of the Mouse Cecum in Early Life
Source: Biology (Basel). 2025 Jul 23;14(8):917. doi: 10.3390/biology14080917 (PMC12383627; doi:10.3390/biology14080917)

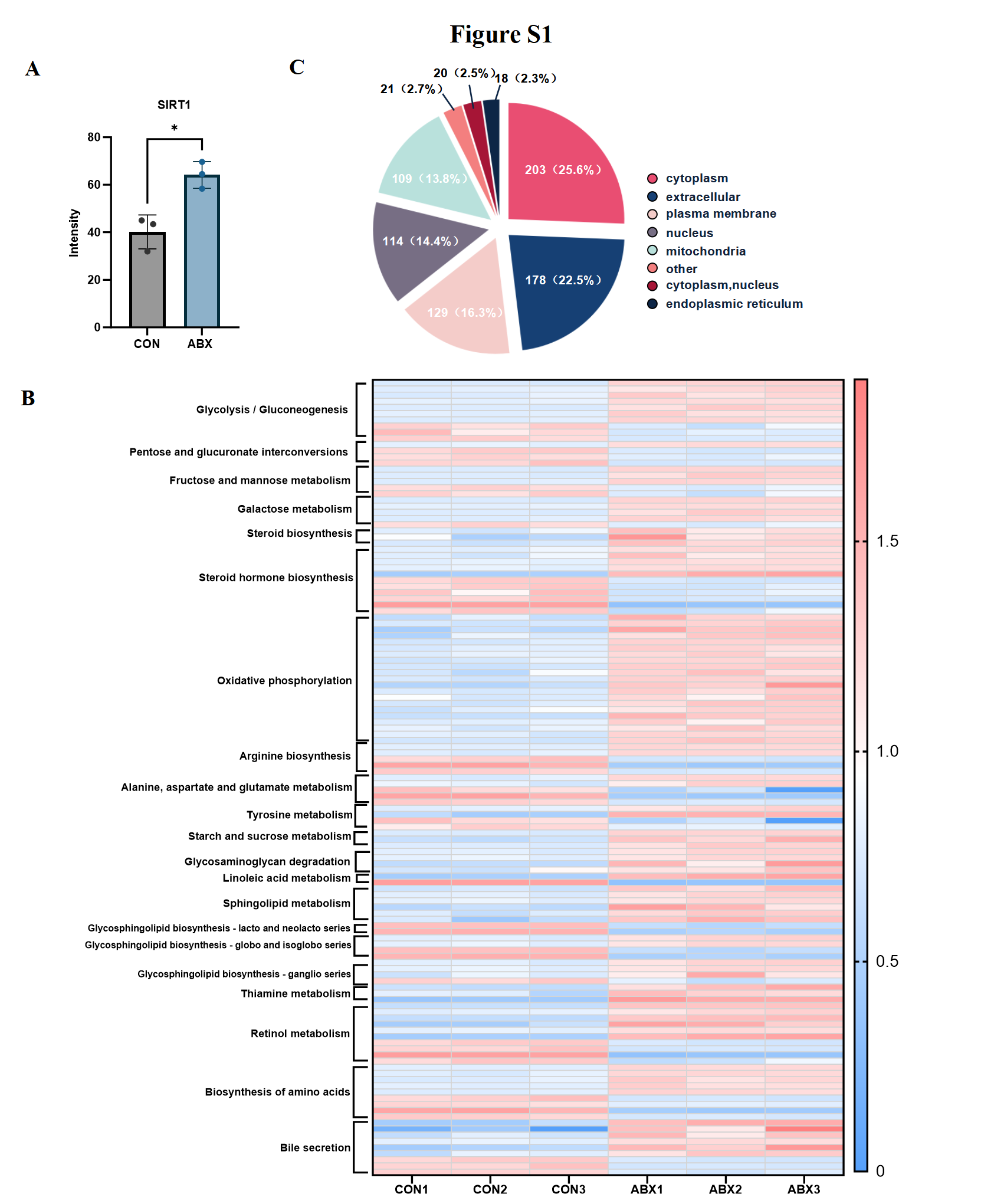

Supplement: Supplementary file 1 [file biology-14-00917-s001.zip › Figure S1.tif]

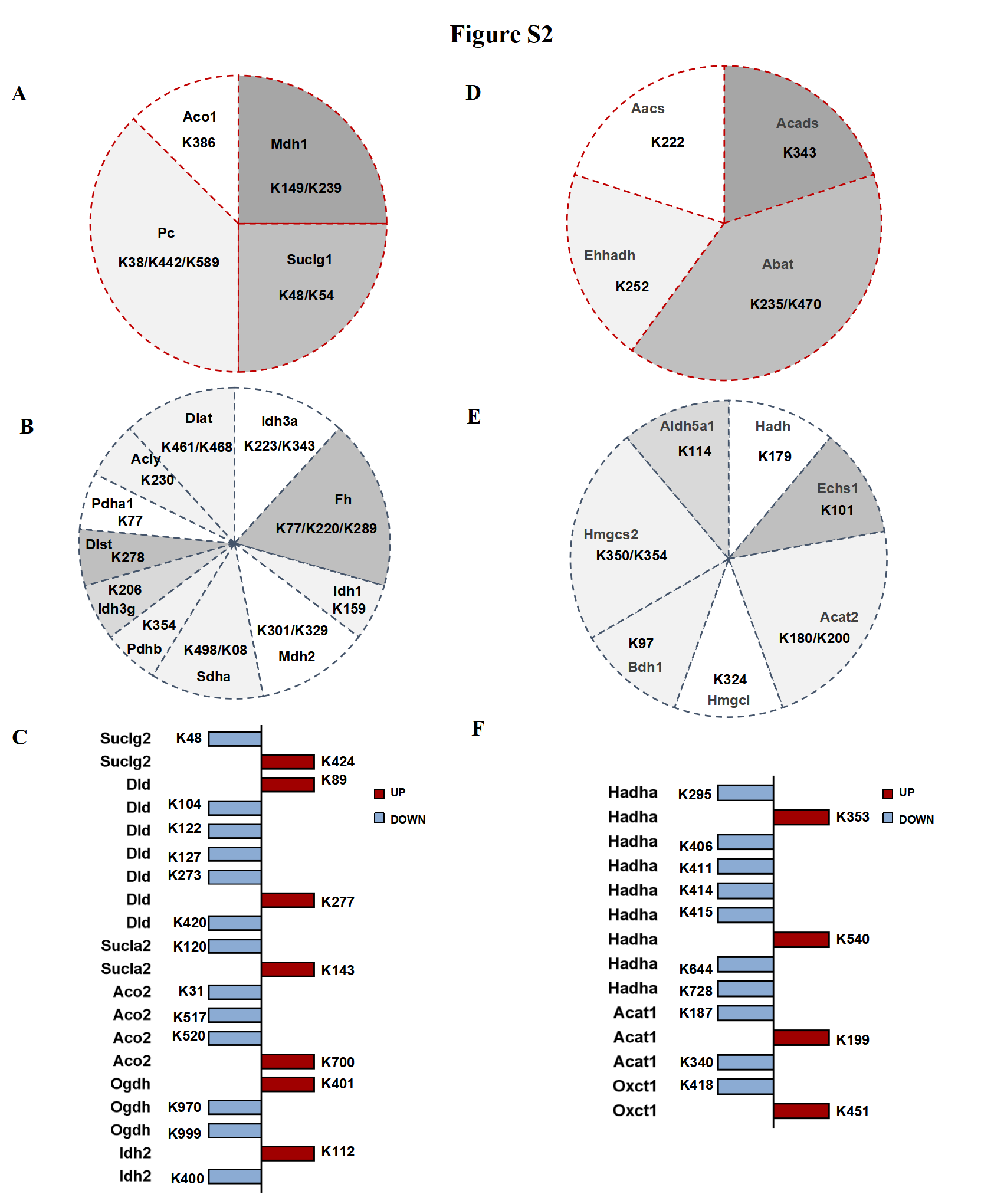

Supplement: Supplementary file 1 [file biology-14-00917-s001.zip › Figure S2.tif]
